# A comprehensive assessment of the malaria microscopy system of Aceh, Indonesia, in preparation for malaria elimination

**DOI:** 10.1186/s12936-015-0746-8

**Published:** 2015-06-11

**Authors:** Lenny L. Ekawati, Herdiana Herdiana, Maria E. Sumiwi, Cut Barussanah, Cut Ainun, Sabri Sabri, Teuku Maulana, Rahmadyani Rahmadyani, Cut Maneh, Muhammad Yani, Paola Valenti, Iqbal R. F. Elyazar, William A. Hawley

**Affiliations:** Paritrana Asia Foundation, Jalan Utama 113, 12640 Jakarta, Indonesia; United Nation Children’s Fund Banda Aceh Zone Office, Jalan Mesjid Shadaqah 2, Lamlagang, 23243 Banda Aceh Indonesia; United Nation Children’s Fund Indonesia, Jalan Jenderal Sudirman Kavling 31, 12920 Jakarta, Indonesia; Aceh Provincial Health Office, Jalan Teungku Syech Mudawali 6, 23242 Banda Aceh, Indonesia; Aceh Provincial Health Laboratory, Jalan Teungku Muhammad Daud Beureuh 168, 23242 Banda Aceh, Indonesia; Eijkman-Oxford Clinical Research Unit, Jalan Diponegoro 69, 10430 Jakarta, Indonesia

**Keywords:** Elimination, Baseline assessment, Microscopy diagnostic, Quality assurance

## Abstract

**Background:**

The Health Office of Aceh aims to eliminate malaria from Aceh Province, Indonesia by 2015. Malaria was formerly common in Aceh (population 4.5 million), but has declined dramatically in recent years consequent to post-tsunami control efforts. Successful elimination will depend upon rapid and accurate diagnosis and case follow-up at community level. A prerequisite to this is widespread coverage of high quality malaria diagnosis. This study describes the results of a comprehensive assessment of the malaria diagnostic capacity in Aceh as the province moves towards malaria elimination.

**Methods:**

The study was conducted in 23 districts in Aceh from October 2010 to July 2011. Six types of questionnaires were used to collect data on competency of microscopists and laboratory capacity. Standardized slides were used to evaluate the proficiency of all microscopists. In addition, site visits to 17 primary health centres (PHC) assessed diagnostic practice and logistics capacity.

**Results:**

Five hundred and seventy four malaria microscopists have been officially registered and assigned to duty in the 23 districts in Aceh Province. They work in 345 laboratories, predominantly in PHCs (69 %) and hospitals (25 %). Three laboratories were evaluated as adequate for all 30 elements, while 29 laboratories were adequate for less than five of 30 elements. Standardized proficiency tests showed that 413 microscopists were at basic (in training) level, with 10 advanced and 9 reference level. No microscopist achieved expert level. Neither the province nor any of Aceh’s districts has a standardized inventory and logistics database for malaria diagnostics, nor did any of the surveyed laboratories operate a quality assurance programme for either microscopy or rapid diagnostic tests.

**Conclusions:**

The study highlights the importance of careful assessment of diagnostic capacity when embarking upon a large-scale malaria elimination programme. Aceh’s laboratories have minimal infrastructure with nearly all microscopists still in training. On the positive side, a large workforce of microscopists has been assigned to laboratories with the needed equipment. Aceh will need to embark on a large-scale comprehensive quality assurance scheme if it is to achieve malaria elimination.

## Background

Successful malaria elimination requires rapid and accurate tracking of cases so that personnel can be promptly deployed to treat cases before onward transmission occurs. With over three million people of its 4.5 million population at risk for malaria transmission, Aceh Province, the westernmost of Indonesia’s provinces, faces a daunting challenge if it is to achieve malaria elimination by its target date of 2015 [1]. From 2005 to 2009, after the surveillance system was revitalized following the Indian Ocean tsunami of 26 Dec 2004, the passive surveillance system detected 6,481 presumptive and 2,355 confirmed malaria cases [2]. The mean prevalence of malaria as measured by 434 small scale malaria surveys conducted from 1985 to 2010 was 2.3 % for *Plasmodium falciparum* (range 0–43 %) and 1.4 % for *Plasmodium vivax* (range 0–31 %) [3, 4]. In 2009 and 2010, the Indonesia Ministry of Health and Aceh Provincial Government launched a call to eliminate malaria. Both provincial and national plans proposed that indigenous transmission of malaria be eliminated from the entire province of Aceh by the end of 2015 [5, 6]. However, a formal assessment of the capacity within the province to eliminate malaria has never been done. High quality malaria diagnostics is acknowledged as a crucial element of successful malaria elimination [7, 8], with microscopy as the gold standard playing a key role [9, 10]. Experience from malaria-free countries has demonstrated the importance of well-established, wide-spread, high quality malaria diagnosis, high capacity for active case follow-up and a robust monitoring and evaluation system [11–13]. Furthermore, extensive experience from countries striving to eliminate malaria in Africa [14–19], Asia-Pacific [1, 20–24] and Peru in South America [25, 26] have shown the necessity of access to reliable and effective diagnosis with microscopy which rests upon the basis of highly trained, well-supervised laboratory technicians who have functioning microscopes, access to re-supply of reagents, respect for positive and negative diagnosis and constant access to anti-malarials if malaria transmission is to be interrupted.

A baseline assessment aims to appraise the strength and weakness, as well as to identify opportunities and challenges of malaria microscopy diagnostics in Aceh Province as it moves toward malaria elimination. Distribution and proficiency of malaria microscopists, laboratories evaluations and observations on the logistics chain and current implementation of quality assurance for microscopy diagnostics were plotted. In overall, the work provides a comprehensive real life example of the variety of challenges faced when a large tropical province moves toward malaria elimination.

## Methods

### Study location

**Fig. 1:**
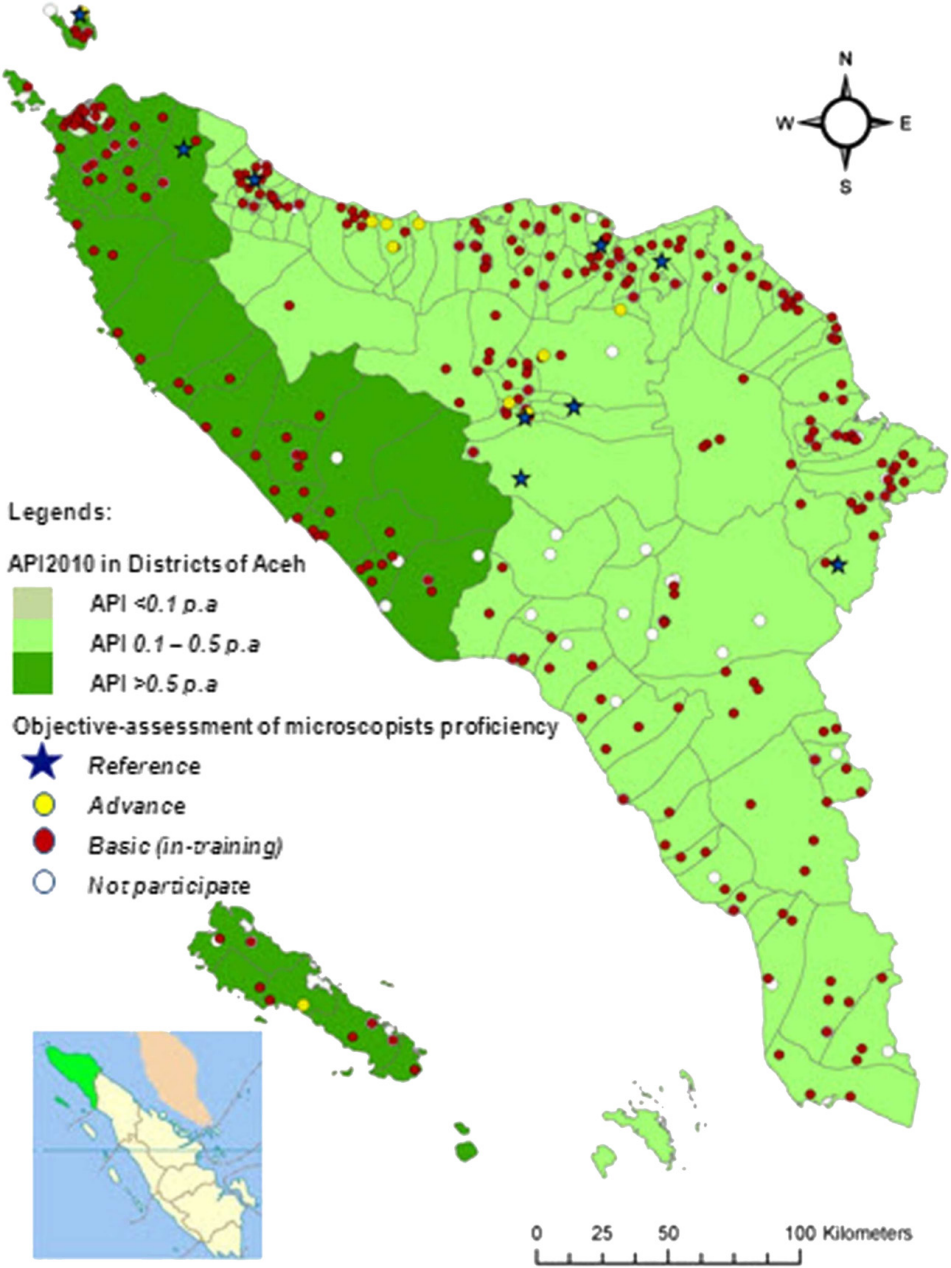
Distribution of malaria microscopists in Aceh province, 2010 - 2011

Aceh (population 4.5 million) is one of 34 provinces in the Indonesian archipelago which occupies 58,375.63 km^2^ on the western tip of Sumatra [27]. Aceh has 23 districts, 276 sub-districts and 6,592 villages [28] with 22 hospitals and 307 primary health centres providing health services to the population [2]. Figure 1 shows map of Aceh and the distribution of microscopists. Participants in the surveys were malaria microscopists working at primary health centres (PHCs), hospitals, government laboratories and health offices in all districts and in the provincial government.

### Assembling the database of malaria microscopists and their laboratories

Two standardized questionnaires were distributed to all PHCs, hospitals and clinics by mail and fax between October and December 2010. The first questionnaire collected personal information on microscopists, such as name, age, gender, educational level, working institution, workload and self-assessment of their diagnostic competency. The second questionnaire aimed to collate perceptions of microscopists about their working conditions, including workspace, equipment, supplies, laboratory, standard operating procedures, human resources, training, safety, collection and data reporting. Questionnaires were returned to provincial health office by mail or fax.

### Evaluating the proficiency of malaria microscopists

Following data assembly, twelve assessors from the Aceh provincial health office (PHO) and provincial health laboratory (PHL) were divided into four groups and assigned to visit 23 districts to carry out on-site proficiency tests. Between May and June 2011, all malaria microscopists were invited for evaluation, with over 400 attending testing at district health offices using standardized malaria slides [29]. Each set consisted of 20 slides containing ten negatives and ten positives consisting of three *P. falciparum*, three *P. vivax*, one *Plasmodium malariae*, one *Plasmodium ovale* and two mixed infection slides. Another set comprised five slides of *P. falciparum* with low to high parasitaemia density for parasite counting. All participants were requested to count for a duration not to exceed ten minutes per slide [30, 31]. They were instructed to detect the presence of malaria parasites from the top left-hand part of the thick smears and move approximately five fields to the right [National Malaria Control Program, personal communications]. They also were instructed to count parasites against 200 leucocytes for parasite density estimates [32]. They were expected to complete the test within four hours and ten minutes. Six diagnostic proficiency indicators, including sensitivity, specificity, overall and species accuracy, error rate and counting agreement were then calculated. Each participant was subsequently graded as in-training, advanced, reference and expert according to the World Health Organization (WHO) guidelines [33].

### Evaluating the practice of malaria diagnostic and logistic

Four districts (Aceh Besar, Aceh Jaya, Aceh Timur and Sabang) were selected for direct observation (confirmatory visits) of malaria diagnostic practice and laboratory conditions. The 64 PHCs in these districts were categorized by slide positivity rate as low (SPR < 5 %), moderate (5 % ≤ SPR < 40 %) and high (SPR ≥ 40 %). For each category, 30 % of PHCs were randomly selected for a confirmatory visit. Six evaluators from the PHO, PHL and an independent malaria quality assurance professional visited the selected PHCs between June and July 2011.

An additional four questionnaires sought information from heads of PHCs, microscopists and logistic officers. The head of PHC questionnaire consisted of 17 questions on administration, case finding, procedure of malaria slide examination and the reporting system. The microscopist questionnaire focused on administration, slide production, diagnosis and laboratory infrastructure, while a subsidiary reporting form was used to record the daily practice of microscopy diagnosis from finger prick procedure to reporting. A fourth questionnaire aimed at logistics officers asked about supply availability, planning, purchasing, storage and distribution of supplies related to malaria diagnosis.

### Statistical analysis

Questionnaire data were entered into an Excel database and then analysed by STATA version 9. Descriptive statistics were calculated for personal information of microscopists and malaria laboratory elements. For each element, an adequacy score was obtained by dividing the number of respondents answering “adequate” by the total number of respondents providing a response. Elements for which ≥ 70 % of respondents responded as adequate were considered as high adequacy and elements for which the score was < 70 % were considered as low adequacy. Six diagnostic proficiency indicators, including sensitivity, specificity, diagnosis accuracy, species accuracy, error rate and parasitaemia counting, were calculated. Sensitivity and specificity were determined as the proportion of parasites detected in standardized positive smears and the proportion with no malaria parasites seen in the standardized negative smears, respectively. Diagnostic accuracy was calculated by dividing the number of slides with correct diagnosis by the total slides examined. Species accuracy was calculated as the proportion of malaria microscopists that accurately determined the species of malaria parasites. Error rate was calculated as the proportion of slides incorrectly diagnosed. Lastly, parasitaemia counting was the proportion of malaria microsopists reporting the number of parasites relative to 200 leucocytes. The density of parasites per microlitre was calculated using the formula: (number of parasites x 8,000 leucocytes)/200 leucocytes. An answer considered correct was obtained when the percentage of quantification was within 25 % of the actual density per microlitre. Kruskal-Wallis rank-test was used to detect the statistical differences between diagnostic proficiency indicators and attendance at examination sessions. Chi-square test was used to assess the proportional differences with a P-value of less than 0.05 indicating a significant difference.

## Results

### Characteristics of malaria microscopists in Aceh

In 23 districts of Aceh, 574 microscopists were registered. All were sent questionnaires with 463 (81 %) responding. Table 1 shows the characteristics of microscopists. More female microscopists were found compared to male (85 % vs 15 %). Mean age of microscopists was 32 years (range 20 to 50 years). Malaria microscopists had a diploma degree as a health analyst (40 %), a high school certificate (23 %), nursing or midwifery degrees (8 %), or bachelor of public health (3 %). Most worked at PHCs (69 %) or hospitals (25 %).

**Table 1:** Characteristics of malaria microscopists in Aceh, Indonesia, 2010

Variables	PHC^a^		Hospital^b^		DHL/O^c^		PHL/O^d^		Total	
	(n = 293)		(n = 34)		(n = 12)		(n = 2)		(n = 341)	
Gender										
Female	340	59.2 %	124	21.6 %	19	3.3 %	6	1.0 %	489	85.2 %
Male	54	9.4 %	23	4.0 %	4	0.7 %	4	0.7 %	85	14.8 %
Total	394	68.6 %	147	25.6 %	23	4.0 %	10	1.7 %	574	100.0 %
Age										
20– < 35 year	233	40.6 %	76	13.2 %	11	1.9 %	2	0.3 %	322	56.1 %
35– < 50 year	85	14.8 %	25	4.4 %	4	0.7 %	6	1.0 %	120	20.9 %
>50 year	0	0.0 %	1	0.2 %	0	0.0 %	1	0.2 %	2	0.3 %
No information	76	13.2 %	45	7.8 %	8	1.4 %	1	0.2 %	130	22.7 %
Total	394	68.6 %	147	25.6 %	23	4.0 %	10	1.7 %	574	100.0 %
Education										
Bachelor	7	1.2 %	5	0.9 %	1	0.2 %	3	0.5 %	16	2.8 %
Diploma	155	27.0 %	63	11.0 %	9	1.6 %	0	0.0 %	227	39.5 %
High school	102	17.8 %	23	4.0 %	4	0.7 %	4	0.7 %	133	23.2 %
Nursing school	39	6.8 %	0	0.0 %	0	0.0 %	0	0.0 %	39	6.8 %
Midwives school	6	1.0 %	0	0.0 %	1	0.2 %	0	0.0 %	7	1.2 %
Other	9	1.6 %	8	1.4 %	0	0.0 %	2	0.3 %	19	3.3 %
No information	76	13.2 %	48	8.4 %	8	1.4 %	1	0.2 %	133	23.2 %
Total	394	68.6 %	147	25.6 %	23	4.0 %	10	1.7 %	574	100.0 %
Length of Service										
<1 year	72	12.5 %	11	1.9 %	4	0.7 %	0	0.0 %	87	15.2 %
1–10 year	195	34.0 %	66	11.5 %	9	1.6 %	3	0.5 %	273	47.6 %
>10 year	48	8.4 %	12	2.1 %	1	0.2 %	3	0.5 %	64	11.1 %
No information	79	13.8 %	58	10.1 %	9	1.6 %	4	0.7 %	150	26.1 %
Total	394	68.6 %	147	25.6 %	23	4.0 %	10	1.7 %	574	100.0 %
Workload										
Mild	285	49.7 %	82	14.3 %	12	2.1 %	8	1.4 %	387	67.4 %
Moderate	9	1.6 %	5	0.9 %	2	0.3 %	0	0.0 %	16	2.8 %
Heavy	3	0.5 %	1	0.2 %	0	0.0 %	0	0.0 %	4	0.7 %
No information	97	16.9 %	59	10.3 %	9	1.6 %	2	0.3 %	167	29.1 %
Total	394	68.6 %	147	25.6 %	23	4.0 %	10	1.7 %	574	100.0 %
Trainings										
Never	107	18.6 %	65	11.3 %	3	0.5 %	0	0.0 %	175	30.5 %
Once	128	22.3 %	16	2.8 %	5	0.9 %	4	0.7 %	153	26.7 %
Twice	56	9.8 %	7	1.2 %	4	0.7 %	2	0.3 %	69	12.0 %
More than three	23	4.0 %	3	0.5 %	2	0.3 %	3	0.5 %	31	5.4 %
No Response	80	13.9 %	56	9.8 %	9	1.6 %	1	0.2 %	146	25.4 %
Total	394	68.6 %	147	11.3 %	23	4.0 %	10	1.7 %	574	69.5 %
Self-proficiency^e^										
Basic	159	27.7 %	58	10.1 %	6	1.0 %	4	0.7 %	227	39.5 %
Intermediate	127	22.1 %	26	4.5 %	1	0.2 %	1	0.2 %	155	27.0 %
Expert	2	0.3 %	0	0.0 %	4	0.7 %	4	0.7 %	10	1.7 %
No information	106	18.5 %	63	11.0 %	12	2.1 %	1	0.2 %	182	31.7 %
Total	394	68.6 %	147	25.6 %	23	4.0 %	10	1.7 %	574	100.0 %
Proficiency^f^										
Basic	314	54.7 %	84	14.6 %	11	1.9 %	4	0.7 %	413	72.0 %
Advance	6	1.0 %	1	0.2 %	3	0.5 %	0	0.0 %	10	1.7 %
Reference	5	0.9 %	0	0.0 %	4	0.7 %	0	0.0 %	9	1.6 %
Expert	0	0.0 %	0	0.0 %	0	0.0 %	0	0.0 %	0	0.0 %
Not assessed	69	12.0 %	62	10.8 %	5	0.9 %	6	1.0 %	142	24.7 %
Total	394	68.6 %	147	25.6 %	23	4.0 %	10	1.7 %	574	100.0 %

^a^ *PHC* Primary Health Centre

^b^ *Hospital* Hospital (*n* = 33) plus Port Office (*n* = 1)

^c^ *DHL/O* District Health Laboratory and District Health Office

^d^ *PHL/O* Provincial Health Laboratory and Provincial Health Office

^e^Proficiency by self-assessment method

^f^Proficiency by objective assessment method

Almost half of microscopists had been carrying out their duties for at least one year and less than 10 years, with about an additional 15 % working less than one year and 11 % for more than 10 years. Most (67 %) examine less than 40 malaria slides per day. Two thirds received at least one microscopy training during their career, with one third receiving no specific training. In terms of duration of service, 11 % of those in service less than one year had no training, 20 % of those in service 1–10 years had no training, whilst 5 % of microscopists working for more than 10 years had never received formal training. Of the 392 microscopists that self-ranked their malaria diagnostic expertise, 57.9 % rated their expertise as basic, followed by 39.5 % as intermediate skill and 2.6 % as experts.

### Features of malaria laboratories in Aceh

Table 2 shows the attributes of malaria laboratories as reported by malaria microscopists in 23 districts in Aceh. Three malaria laboratories – the Provincial Health Laboratory in Banda Aceh, PHC Bandar Dua, and PHC Bandar Baru in Pidie Jaya district reported adequacy at all elements. Two districts, Gayo Lues and Simeuleu, provided no response regarding capacity of their malaria laboratories. Laboratories in six districts reported at least 20 adequate elements, including Pidie Jaya, Sabang, Banda Aceh, Pidie, Aceh Tamiang and Lhokseumawe. The majority of malaria microscopists reported high adequacies for certain components, including a clean and tidy room, appropriate storage of equipment, adequate stocks of supplies, efficient workflow, clear laboratory procedures and effective slide archiving. Microscopists in Aceh Barat, Aceh Timur and Nagan Raya districts reported low adequacy for training documentation, no regular quality checks on supplies, lack of maintenance records for equipment, no annual review on laboratory SOPs, lack of a cross-checking system and failure to limit access of unauthorized personnel to the laboratory.

**Table 2:** Features of malaria laboratories in Aceh, Indonesia, 2010

Laboratory Components	Aceh Barat	Abdiya	Aceh Besar	Aceh Jaya	Aceh Selatan	Aceh Singkil	Aceh Tamiang	Aceh Tengah	Aceh Tenggara	Aceh Timur	Aceh Utara	Banda Aceh	Bener Meriah	Bireun	Gayo Lues	Langsa	Lhokseumawe	Nagan Raya	Pidie	Pidie Jaya	Sabang	Simeuleu	Subulussalam
Number of Laboratories	13	14	24	10	13	10	16	19	14	29	28	23	12	22	14	9	8	11	20	10	10	10	5
Working space																							
Facility and space	●	o	●	●	●	●	●	●	●	o	●	●	●	●	■	●	●	o	●	●	●	■	●
Limited access	o	●	o	o	o	o	o	●	o	o	●	o	●	o	■	o	o	o	●	o	o	■	●
Clean and tidy room	●	o	●	●	●	●	●	●	●	●	●	●	●	●	■	o	●	●	●	●	●	■	●
Water supply	o	o	●	o	o	●	●	●	o	o	o	●	o	●	■	●	●	●	●	o	●	■	●
Electricity	●	●	●	●	●	o	●	●	o	●	●	o	o	●	■	o	o	●	●	●	●	■	●
Equipment																							
Availability	o	o	●	●	●	●	●	o	o	o	●	●	●	●	■	●	o	o	●	●	●	■	●
Maintenance records	o	o	o	o	●	o	o	o	o	o	o	●	o	o	■	●	o	o	o	●	o	■	o
Calibration	o	o	o	o	●	o	o	●	o	o	o	●	o	o	■	●	o	o	o	●	o	■	o
Storage	●	o	●	●	●	●	●	o	●	o	●	●	●	●	■	●	●	●	●	●	●	■	●
Supplies																							
Stock of supplies	o	o	●	●	●	●	●	●	●	o	●	●	●	●	■	●	●	o	●	●	●	■	●
Quality check	o	o	o	o	o	o	●	o	o	o	o	●	o	o	■	o	o	o	●	●	o	■	o
Procurement	o	o	●	●	o	o	●	●	o	o	●	o	●	●	■	o	●	o	o	●	o	■	●
Laboratory																							
Efficient workflow	●	o	●	●	●	o	●	●	●	o	●	●	●	●	■	●	●	o	●	●	●	■	●
Register book	●	o	●	●	●	o	●	●	●	o	●	●	●	●	■	o	●	o	●	●	●	■	●
Information updated	●	o	●	o	●	o	●	●	●	o	●	●	o	●	■	o	●	o	●	●	●	■	●
SOPs																							
Laboratory procedures	●	o	●	●	●	●	●	●	●	o	●	●	●	●	■	●	●	o	●	●	●	■	o
Manual for equipment	●	o	●	●	●	o	●	●	●	o	●	●	●	●	■	●	●	o	●	●	●	■	o
SOP’s location	o	o	o	●	●	o	●	●	o	o	●	●	o	●	■	●	●	o	●	●	●	■	o
Reflect current work	●	o	o	●	●	o	●	●	o	o	●	o	●	o	■	●	●	o	●	●	●	■	o
Annually reviewed	o	o	o	●	o	o	o	o	o	o	o	●	o	o	■	●	o	o	o	●	●	■	o
HR and Trainings																							
Microscopists	●	o	●	●	o	●	●	o	●	●	●	●	●	o	■	●	●	o	●	●	●	■	●
Refresher training	o	o	o	o	o	●	●	o	o	●	o	●	●	o	■	o	●	o	o	●	●	■	●
Training documents	o	o	o	o	o	o	o	o	o	o	o	o	o	o	■	●	o	o	o	o	●	■	o
Microscopy trainings	●	o	o	o	o	o	o	o	o	●	o	●	●	o	■	●	o	o	o	●	●	■	●
Collection and reporting																							
Slides archiving	●	●	●	●	●	o	●	o	●	o	●	●	●	●	■	●	●	o	●	●	●	■	●
Reporting	●	●	●	●	o	o	●	o	●	o	●	●	●	●	■	o	●	●	●	●	●	■	●
Cross-checking	o	o	o	●	o	o	o	o	o	o	o	o	●	o	■	●	●	o	o	●	o	■	o
Safety																							
Sharp container	●	o	●	●	●	●	o	●	o	●	o	●	o	●	■	●	●	●	●	o	●	■	o
Laboratory coat	●	●	o	o	o	o	o	o	o	o	o	o	o	●	■	o	●	o	●	●	●	■	o
Handwash soap	●	o	●	o	●	●	●	●	o	o	o	●	●	●	■	o	●	o	●	o	●	■	o

●: High adequacy; score ≥ 70 %

o: Less adequacy, score < 70 %

**■**: No response

**Fig. 2:**
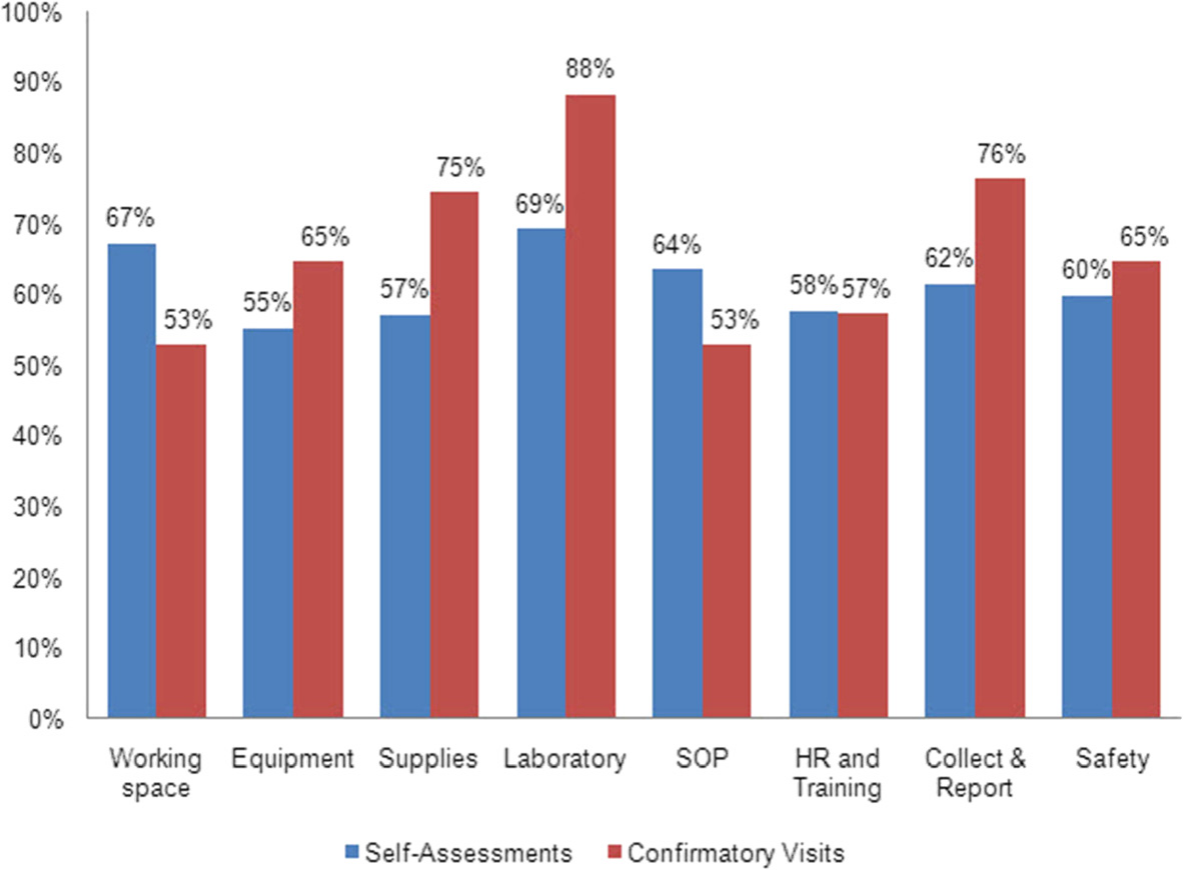
Comparison of proportion of malaria laboratory elements rated by self-assessment and direct observation in Aceh, Indonesia, 2010 – 2011

Results of self-assessments and confirmatory visits were broadly similar as shown in Fig. 2, which shows the proportion of laboratories self-reporting as adequate versus results of external assessment for 17 PHCs and eight elements. No significant differences were detected in adequacy rates for any of the eight indicators, which increase a confidence that self-reporting for these elements accurately reflects reality. Results of self-assessment and field visits both reported inadequate laboratory conditions. Table 3 shows results of field visits to 17 PHCs in four districts. Out of 30 elements examined, low adequacy was found in 22 elements in Aceh Besar, 20 elements in Aceh Timur, 16 in Aceh Jaya, with Sabang the only district demonstrating low adequacy in less than half (13) of the elements examined. Assessors commonly observed unrestricted access to laboratories, lack of manuals for equipment, poor supply quality control checks, uncommonly applied annual review of working procedures, and infrequent refresher training. Most PHCs in Aceh Besar, Aceh Jaya and Aceh Timur were unlikely to have equipment maintenance records or calibration, storage and safety procedures, such as proper sharp containers, laboratory coats and handwashing soaps. Both Aceh Besar and Aceh Timur districts lacked adequate working space and clean water as shown in Fig. 2. With the exception of two PHCs in Sabang, no cross-checking systems had been implemented.

**Table 3:** Characteristics of malaria diagnostic laboratories by self-assessment and direct confirmation visits at 17 PHCs in four districts in Aceh, 2011

	Districts							
Laboratory Components	Aceh Besar (n = 6)		Aceh Jaya (n = 3)		Aceh Timur (n = 6)		Sabang (n = 2)	
	S	C	S	C	S	C	S	C
Working space								
Facility and space	●	o	o	●	●	o	●	●
Limited access	o	o	o	o	o	o	o	o
Clean and tidy room	●	●	o	●	●	●	●	●
Water supply	o	o	o	●	o	o	●	●
Electricity	o	●	●	●	●	●	●	●
Equipment								
Availability	o	o	o	o	o	o	●	o
Maintenance records	o	o	o	o	o	o	o	o
Calibration	o	o	o	o	o	o	o	●
Storage	●	●	o	●	●	●	●	●
Supplies								
Stock of supplies	o	●	o	●	o	●	o	●
Quality check	o	o	o	o	o	o	o	o
Procurement	o	●	o	o	o	●	o	●
Laboratory								
Efficient workflow	o	o	●	●	●	●	●	●
Register book	●	●	●	●	●	o	●	●
Information updated	o	o	o	●	●	o	●	●
SOP								
Laboratory procedures	●	o	o	●	●	o	●	o
Manual for equipment	o	o	o	o	●	o	●	o
SOP’s location	o	●	o	o	o	o	●	o
Reflected current work	o	o	o	●	o	o	●	o
Annually reviewed	o	o	o	o	o	o	●	o
HR and Trainings								
Microscopists	o	o	o	●	●	o	●	●
Refresher training	o	o	o	o	●	●	o	o
Training documents	o	o	o	o	o	o	●	o
Microscopy trainings	o	o	●	o	●	●	●	o
Collection and reporting								
Slides archiving	o	o	o	●	●	●	●	●
Reporting	●	●	●	●	●	●	●	●
Cross-checking	o	o	●	o	●	o	●	●
Safety								
Sharp container	●	o	●	o	●	o	●	●
Laboratory coat	o	o	●	o	o	o	●	o
Handwash soap	●	o	o	o	o	o	●	●

S: Self-assessment C: Confirmatory-check

●: High adequacy; score ≥ 70 %

o: Less adequacy; score < 70 %

### Proficiency of malaria microscopists

Four hundred thirty two microscopists of 574 accepted invitations to participate in proficiency testing. Nearly all microscopists (413 or 95.6 %) scored at basic or in-training level, while 10 (2.3 %) were advanced and 9 (2.1 %) were reference microscopists. Among the ten advanced microscopists, six worked at PHCs, three at district level (two at health offices, one at health laboratory) and one at a hospital. The advanced microscopists were deployed at Bireun (3), Bener Meriah (2), Aceh Tengah (1), Aceh Utara (1), Pidie Jaya (1), Sabang (1) and Simeuleu (1). Among nine reference microscopists, five worked at PHCs and four at district health offices. These reference miscroscopists were located in Aceh Tengah (3), Aceh Besar (1), Aceh Tamiang (1), Aceh Utara (1), Bireun (1), Pidie (1) and Sabang (1) (Fig. 1).

Microscopists who reported having had microscopy training performed significantly better than those who reported no training for diagnosis accuracy (70.2 *vs* 60.8 %, *p* < 0.001), sensitivity (69.1 *vs* 57 %, *p* < 0.001), species accuracy (37.2 % *vs* 27.7 %, *p* < 0.001) and parasitaemia counting (25 % *vs* 19.8 %, *p* = 0.04), but they did not perform significantly better for specificity (70.6 *vs* 65.3 %, *p* = 0.115).

### The practice of malaria diagnostic at primary health centres

Sixteen heads of PHCs (eight of whom were clinicians) were interviewed during confirmatory visits; all confirmed that their PHC provided free microscopy diagnostic services. Ten of 16 heads of PHCs referred patients for laboratory examination on the basis of recent fever, history of malaria infection, or travel to malaria endemic areas. Only one respondent said that laboratory malaria diagnosis was unnecessary in areas where malaria was found rarely (1/16).

Most (14/16) heads of PHCs were satisfied with their microscopist’s performance, believing them to be competent (12/16), properly trained (11/16), properly archiving slides (12/16), supported by a cross check system (13/16), and having adequate equipment, laboratory supplies and storage space (12/16). Half (8/16) of clinicians reported receiving malaria results within less than an hour. Slightly over half (9/16) were aware of the absence of written standard operational procedures for laboratories (9/16). Clinicians in Aceh Timur complained that it takes 12–24 h to get confirmed results. In consequence, they treated patients presumptively.

**Fig. 3:**
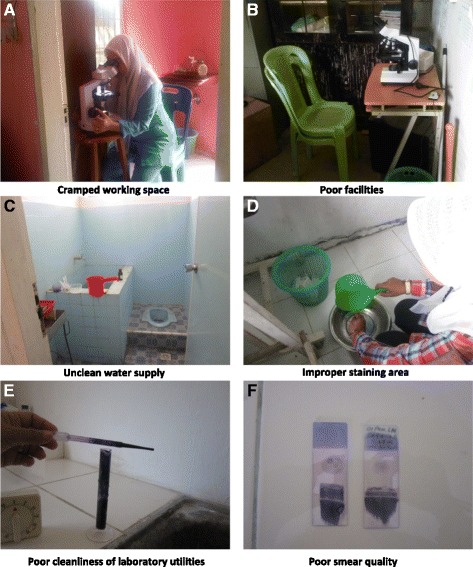
Less than adequate laboratories in Aceh, 2011

Figure 3 and Table 4 show the results of field visits to assess microscopy diagnostic practices of 25 microscopists at PHCs. Microscopists in Aceh Besar and Aceh Timur were less capable than their counterparts in Aceh Jaya and Sabang. Most microscopists used non-frosted-end object slides making it difficult to record patient identifiers directly on the slides. In many cases, laboratory space and equipment were not properly cleaned, possibly increasing the number of artefacts from staining and increasing the error rate of microscopists. The quality of thick and thin smears produced was generally low, and most microscopists examined fewer than the recommended 200 fields before determining that a slide is negative. No microscopist calculated parasite density, with qualitative measures (+, ++, +++) used instead, calling into question their ability to detect low-density parasitaemia.

**Table 4:** Major findings in the procedures of microscopy diagnosis obtained from supervisory visits to 17 PHCs in four districts of Aceh, 2011

Microscopy Diagnostic Components	Districts			
	Aceh Besar	Aceh Jaya	Aceh Timur	Sabang
Preparation:				
Cleaned object glasses	●	o	●	●
Frosted-end object glasses	o	●	●	●
Patients identities	o	o	o	o
Laboratory utilities	o	o	o	o
Making malaria smears:				
Blood volume	o	o	o	●
Quality of smears production	o	o	o	o
Staining:				
Fixation	●	●	o	●
Materials and supplies	o	o	o	o
Composition of Giemsa and buffer	●	●	o	●
Quality smears after staining	●	o	o	●
Quality control for reagents	o	o	●	●
Slide examination:				
Using microscope	o	o	o	●
Observe 200 fields	o	o	o	o
Time consuming (>10 min)	o	●	o	●
Clear from artefacts	o	o	o	o
Counting:				
Counting procedures	●	o	●	o
Count at least 200 Leucocytes	o	o	o	o
Never report parasites density	o	o	o	o
Counting more than 10 min	o	●	o	●
Quality assurance				
Slides archiving	o	●	o	●
Cross-checking	o	o	o	●

●: Adequate, refresher training not required

o: Inadequate, refresher training is recommended

### Malaria diagnostics logistic chain

**Fig. 4:**
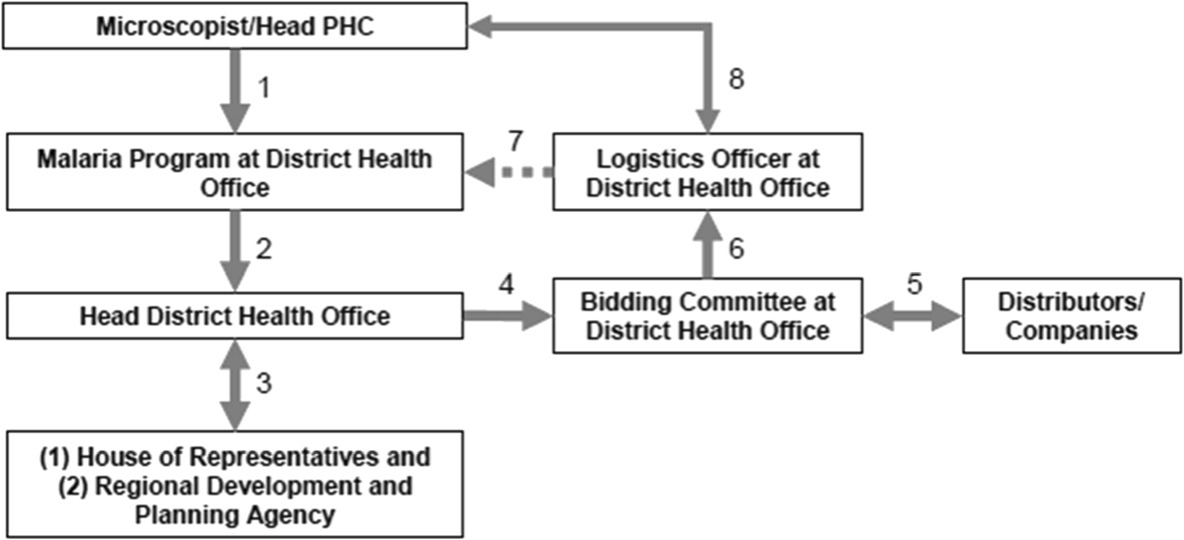
Scheme of logistics and purchasing system in microscopy diagnostic of malaria in Aceh province, 2010 - 2011

Figure 4 outlines the administrative and bureaucratic processes involved in purchasing logistics for malaria diagnostics in Aceh. Microscopists work through the heads of their PHCs, who submit plans for budget review at the district. Consolidated plans are then presented to the District Parliament and District Planning Board (Bappeda, *Badan Perencanaan Pembangunan Daerah*) for final approval. Approval does not end the process, however, as the district purchasing committee must then open the bidding process, contact vendors, and manage procurement. When materials arrive, logistics officers inventory and store the supplies in the district warehouse. Logistics are normally delivered to the PHCs but in some cases PHC staff visited the warehouse to pick up supplies.

Twenty two people responsible for malaria diagnostics logistics at 17 PHCs and four DHOs were interviewed to assess the effectiveness of this supply chain. Fourteen of those interviewed also currently worked as malaria microscopists. Most respondents said that they neither created a purchasing plan (15) nor read guidelines on the purchasing system (16). Two respondents reported that their PHCs planned their annual budget once a year in October.

Most respondents reported that their logistics for malaria diagnosis arrived on time (14). Most respondents reported that they had sufficient storage space for supplies (15) and said that regular inventories to prevent stock outs were done (14). More than half of the respondents reported that it might be useful to have a storage cabinet at temperature ≤30 °C for RDTs, but that it was unlikely the available space was sufficient (12). Some respondents reported that the quantities of supplies received were less than the amount requested (3).

Various frequencies of reporting on reagents and consumables consumption were reported by respondents. The majority reported monthly (11), while others reported quarterly (2), twice a year (1), once a year (1) or never (2).

## Discussion

This study represents one of the few efforts to comprehensively document the readiness of a large administrative unit – in this case, the province of Aceh, Indonesia, with a population of 4.5 million people – to carry out the high quality diagnosis needed for malaria elimination. Thanks to large scale malaria control efforts including LLIN distribution, IRS, and roll-out of ACT in Aceh post-tsunami, the incidence of malaria has declined to the point where mapping and follow-up of individual cases known as “active” case surveillance for malaria elimination has become feasible [34]. However this is premised on a sufficiently robust and effective diagnostic network. Self-assessment was used to ensure wide coverage, coupled with direct observation for selected health facilities and objective assessment of the capacity of most of the microscopists in the province. Some selection bias is possible as 75 % of those sent questionnaires responded. A database for 574 microscopists providing diagnostic services in 293 PHCs, 33 hospitals, 12 district health offices, four district health laboratories, one port health office, one provincial health office and one provincial health laboratory was assembled. The objective-proficiency tests revealed that nearly two thirds of microscopists in the province graded as basic level according to the WHO standard [33]. It seems unlikely that those not tested would be of higher competency that those tested, so think it likely that the overall competence of Aceh microscopists is at a basic level.

The results of the assessment showed that microscopists in Aceh tended to overestimate their own competency. One third of surveyed microscopists (36 %) rated their competency as “intermediate” or “expert”, even though they had never attended microscopy training. Seventy-four percent of 155 self-described ‘intermediate’ microscopists tested at basic level, whereas three self-assessed ‘expert’ microscopists tested as basic, advanced, and reference, respectively. Objective assessment of microscopist’s competency using standardized slides is essential in this context, given the severe bias of self-assessments to overestimate competency and simple one-off training will not ensure diagnostic quality [35]. In contrast, the assessment of microscopists of their working environment and laboratory infrastructure closely mirrored the objective assessment of the independent team’s direct observations.

The assessment demonstrated that the microscopists in Aceh are not sufficiently skilled to detect the low-density parasitaemia associated with malaria elimination efforts; such ability is key in low transmission areas such as Aceh [36–38]. Only expert microscopists with adequate experience are likely able to detect and quantify low parasite densities [10]. However, simple one-off training will not ensure diagnostic quality [35]. For instance, in East Aceh district 25 people received standard microscopy training in 2010 organized by UNICEF and the Aceh Provincial Health Office [39]. When retested 10 months post-training, there had been significant erosion of diagnostic accuracy skills (62.4 *vs* 82.7 %, *p* < 0.001), sensitivity (73.6 *vs* 92 %, *p* < 0.001) and specificity (51.2 *vs* 66.6 %, *p* = 0.032). In the context of malaria elimination, false negative finds are particularly inimical. In addition, visits to facilities showed that microscopists were not supported by adequate infrastructure (Table 3). A study in Thailand emphasized that adequate microscope and quality control procedures were crucial [20], as well as supervisors’ training to conduct visits and evaluate the laboratories [31].

The effectiveness of a rigorous training and supervisory policy has been shown on the Thai-Myanmar border, where microscopists with objective accuracy of more than 80 % are assigned to work in PHCs [20]; those less competent are required to attend competency-based training [31]. Such intense effort is in part due to substantial funding available to reduce anti-malarial drug resistance in the Mekong region. Microscopists with marginal scores of 70–80 % accuracy undergo additional training and close supervision for six months. No such policy for competency improvement yet exists in Aceh, nor is there global interest, so that local initiatives will need to prevail.

The WHO recommends that at least one well-trained and competent malaria microscopist works in each PHC [9]. To achieve this, Aceh province will need to develop a routine accreditation programme, minimize turnover of malaria microscopists, and provide strong support for its small number of reference microscopists to become experts [40]. A small group of expert microscopists can serve as both cross-checkers and trainers. Training should be individualized, emphasizing the weakest skills for each trainee. For example, microscopists from the district of Aceh Besar require training in slide preparation, slide reading and quality assurance, while microsopists from Aceh Jaya District need more training on slide preparation. In Aceh Timur, the weakest elements were on staining and slide examination. Four districts (Aceh Besar, Aceh Jaya, Aceh Timur and Sabang) need to improve their capability to produce good malaria smears and accurate parasitaemia counts. Adequacy of laboratory facilities is an important influence on the competence of microscopists. Two studies in Africa rated laboratories by several fundamental elements (in addition to having well-trained staff), including reliable electricity, access to clean water, adequate working space, comfortable room temperature, proper venipuncture procedure and routine physician oversight [41, 42]. Using these criteria, most laboratories in Aceh (63 %) were classified as minimal, generally because of unreliable access to electricity and clean water, limited equipment and minimally trained personnel.

Logistics and procurement policy have a large impact on laboratory quality. In Aceh, the system of procurement is initiated by the microscopists, but in most cases guidelines for minimal standards are either not available or not applied. Essential elements are a procurement policy, and guidelines on equipment and reagents specifying minimum standards [31].

Sustained and high quality logistics are essential for high quality malaria microscopy [31, 43]. The responsible logistic or malaria programme officers at all levels of the health system should ensure the quantity and quality of logistics prior to transporting to PHCs [43] and ensure continuous supply [40] of both required diagnostics and treatment requirements. Logistic officers, who are mostly also the malaria microscopists, need training to manage their inventory system, *i.e.*, to create inventory lists, monitor stocks and submit routine reports. This would ensure that they are aware of their logistics requirements and stocks in the medium term (six months) and would allow sufficient advance time in reporting to avoid lapses in supply from the DHO [31]. Such reports would enable logistic officers to predict and avoid stock-outs of microscopy diagnostic supplies [40]. Involving malaria microscopists in the purchasing plan is useful because of their hands-on experience with different supplies.

Implementation of a good External Quality Assessment (EQA) would likely increase the competency of microscopists in Aceh. At present, the cross-checking system adopts a traditional policy to send all positive slides and 5 to 10 % of negative slides to a microscopist at a higher and presumably more competent level. However, the system is barely functional, with few microscopists sending slides for cross-checking and even fewer receiving prompt and useful feedback. Part of the reason for this is the heavy administrative burden and amount of labor associated with the numerous slides submitted. In 2009, the WHO [31] recommended two alternative approaches for random selection of slides for cross-checking at PHCs: Lots Quality Assurance System (LQAS) and the Médecins Sans Frontières (MSF) system. One study in Pakistan showed that a useful LQAS-based cross-check system could be based upon sending only eight per month based on slide positivity rate (SPR) and the number of negative slides in the previous year [24]. Several countries, including Thailand, Philippines, Pakistan and Ghana [24, 30, 31, 44] have implemented this scheme for malaria and tuberculosis. In the MSF method, five negative and five weak positive slides are sent to be re-checked, based upon the assumptions that false positive slides were most likely occurring during routine examination because microscopists preferred to ‘play safe’ and report positive result for true negative slide or because of misidentification of artefacts as parasites [45, 46]. The Aceh Provincial Health Office has decided to begin implementation of LQAS for cross-checking soon.

Establishing an effective quality assurance (QA) system for microscopy diagnosis requires a comprehensive approach. Achieving and maintaining high quality performance of microscopists in various malaria endemicity settings involves multiple interventions, including dissemination of written national guidelines, modern microscopy training using computer-based modules, and supervision and feedback [31, 35, 46]. Intensive performance monitoring is likely more constructive than allowing microscopists to repeatedly attend identical standard training modules [47]. To achieve malaria elimination, Aceh province needs to immediately start implementing a comprehensive large-scale QA system and advocate to decision-makers to recruit proficient malaria microscopists.

## References

[1] Elyazar IRF, Hay SI, Baird JK (2011). Malaria distribution, prevalence, drug resistance and control in Indonesia. Adv Parasitol..

[2] Aceh Provincial Health Office. Health profile in Aceh Province. 2009. p. 86.

[3] Elyazar IRF, Gething PW, Patil AP, Rogayah H, Kusriastuti R, Wismarini DM (2011). *Plasmodium falciparum* malaria endemicity in Indonesia in 2010. PLoS One..

[4] Elyazar IR, Gething PW, Patil AP, Rogayah H, Sariwati E, Palupi NW (2012). *Plasmodium vivax* malaria endemicity in Indonesia in 2010. PLoS One..

[5] Departemen Kesehatan Republik Indonesia (2009). Keputusan Menteri Kesehatan Republik Indonesia Nomor 293/MENKES/SK/IV/2009 Tentang Eliminasi Malaria.

[6] Gubernur A (2010). Peraturan Gubernur Aceh No. 40 Tahun.

[7] WHO (2009). Parasitological confirmation of malaria diagnosis: report of a WHO technical consultation, 6–8 October 2009.

[8] Feachem RGA, Phillips AA, Targett GA (eds). Shrinking the malaria map: a prospectus on malaria elimination. San Francisco: The Global Health Group, Global Health Sciences, University of California, San Francisco, 2009:187. http://www.malariaeliminationgroup.org/publications/shrinking-malaria-map-prospectus-malaria-elimination.

[9] WHO (2007). Malaria elimination: a field manual for low and moderate endemic countries.

[10] The malERA Consultative Group on Diagnoses and Diagnostics (2011). A research agenda for malaria eradication: diagnoses and diagnostics. PLoS Med.

[11] Dowling MAC (1951). An experiment in the eradication of malaria in Mauritius. Bull World Health Organ..

[12] Milne LM, Kyi MS, Chiodini PL, Warhurst DC (1994). Accuracy of routine laboratory diagnosis of malaria in the United Kingdom. J Clin Pathol..

[13] Kettelhut MM, Chiodini PL, Edwards H, Moody A (2003). External quality assessment schemes raise standards: evidence from the UKNEQAS parasitology subschemes. J Clin Pathol..

[14] Hamer DH, Ndhlovu M, Zurovac D, Fox M, Yeboah-Antwi K, Chanda P (2007). Improved diagnostic testing and malaria treatment practices in Zambia. JAMA..

[15] Njama-Meya D, Clark TD, Nzarubara B, Staedke S, Kamya MR, Dorsey G (2007). Treatment of malaria restricted to laboratory-confirmed cases: a prospective cohort study in Ugandan children. Malar J..

[16] Ngasala B, Mubi M, Warsame M, Petzold MG, Massele AY, Gustafsson LL (2008). Impact of training in clinical and microscopy diagnosis of childhood malaria on antimalarial drug prescription and health outcome at primary health care level in Tanzania: a randomized controlled trial. Malar J..

[17] Ishengoma DRS, Rwegoshora RT, Mdira KY, Kamugisha ML, Anga EO, Bygbjerg IC (2009). Health laboratories in the Tanga region of Tanzania: the quality of diagnostic services for malaria and other communicable diseases. Ann Trop Med Parasitol..

[18] Mukadi P, Gillet P, Lukuka A, Atua B, Kahodi S, Lokombe J (2011). External quality assessment of malaria microscopy in the Democratic Republic of the Congo. Malar J..

[19] Kiggundu M, Nsobya SL, Kamya MR, Filler S, Nasr S, Dorsey G (2011). Evaluation of a comprehensive refresher training program in malaria microscopy covering four districts of Uganda. Am J Trop Med Hyg..

[20] Hemme F, Gay F (1998). Internal quality control of the malaria microscopy diagnosis for 10 laboratories on the Thai-Myanmar border. Southeast Asian J Trop Med Public Health..

[21] Desato Y, Mau F: Studi pemastian kualitas (quality assurance) pemeriksaan mikroskopis malaria Puskesmas di Pulau Sumba. Laporan Penelitian Departemen Kesehatan RI Badan Penelitian dan Pengembangan Kesehatan Loka Penelitian dan Pengembangan Pemberantasan Penyakit Bersumber Binatang (LOKAL LITBANG P2B2) 2006:32.

[22] Leung WL, Kam KM (2007). Malaria parasites quality assurance programme in Hong Kong, 2002–2006. Journal Hong Kong Institute of Medical Laboratory Services..

[23] Harris I, Sharrock WW, Bain LM, Gray KA, Bobogare A, Boaz L (2010). A large proportion of asymptomatic Plasmodium infections with low and sub-microscopic parasite densities in the low transmission setting of Temotu Province, Solomon Islands: challenges for malaria diagnostics in an elimination setting. Malar J.

[24] Khan MA (2011). District level external quality assurance (EQA) of malaria microscopy in Pakistan: pilot implementation and feasibility. Malar J..

[25] Roshanravan B, Kari E, Gilman RH, Cabrera L, Lee E, Metcalfe J (2003). Endemic malaria in the Peruvian Amazon Region of Iquitos. Am J Trop Med Hyg..

[26] Aguirre AR, Gamboa D, Rodriguez H, Zavalaga FL, Aguirre K, Cuentas AL (2010). Use of standardized blood smear slide sets for competency assessment in the malaria microscopic diagnosis in the Peruvian Amazon. Rev Peru Med Exp Salud Publica..

[27] The Indonesia Central Bureau of Statistics (2010). Trends of selected socioeconomic indicators in Indonesia.

[28] Aceh Provincial Health Office. Health profile in Aceh Province. 2010. p. 108.

[29] Syafruddin D (2011). Establishment of sustainable high quality of malaria diagnosis in Aceh Province and Sabang District. Final Report to United Nations Children’s Fund (UNICEF) Contract No: SSA/IDSO/2010/00000013-0.

[30] WHO (2005). Malaria light microscopy creating a culture of quality. Report of WHO SEARO/WPRO workshop on quality assurance for malaria microscopy, 18–21 April 2005.

[31] WHO (2009). Malaria microscopy quality assurance manual. Version 1.

[32] WHO (2010). Basic malaria microscopy. Part I. Learner’s guide.

[33] WHO (2006). Informal consultation on quality control of malaria microscopy.

[34] Herdiana H, Fuad A, Asih PB, Zubaedah S, Arisanti RR, Syafruddin D (2013). Progress towards malaria elimination in Sabang Municipality, Aceh. Indonesia. Malar J..

[35] Mills A, Brugha R, Hanson K, McPake B (2002). What can be done about the private health sector in low-income countries?. Bull World Health Organ..

[36] Nanyingi M (2008). Adherence to laboratory findings in the management of malaria in the high and low transmission areas of Nakasongola and Kabalore Districts of Uganda. Health Policy and Development Journal..

[37] Maro KJ, D’Acremont V, Mtasiwa D, Genton B, Lengeler C (2011). Low quality of routine microscopy for malaria at different levels of the health system in Dar es Salaam. Malar J..

[38] Enwuru CP, Umeh SI, Abasi UM, Egbuobi RC (2011). Laboratory diagnosis of malaria in children under five years in a rural community: microscopy versus malaria PF test. Afr J Clin Exp Microbiol..

[39] Ekawati LL, Elyazar IRF, Masbar S, Barusannah C, Daniel T. Training on microscopy diagnostic of malaria for health professionals in primary health centers and hospital in district of East Aceh, Aceh province. Activity Report. 2010:28.

[40] WHO (2011). Achieving universal access to malaria diagnostic testing: an operational manual.

[41] Rafael ME, Taylor T, Magill A, Lim YW, Girosi F, Allan R. Reducing the burden of childhood malaria in Africa: the role of improved diagnostics. Nature. 2006:39–48. 10.1038/nature05445 17159893

[42] Urdea M, Penny LA, Olmsted SS, Giovanni MY, Kaspar P, Shepherd A (2006). Requirements for high impact diagnostics in the developing world. Nature..

[43] Zanzibar Malaria Control Programme (2009). Malaria elimination in Zanzibar: a feasibility assessment.

[44] Association of Public Health Laboratories (APHL) (2002). External quality assessment for AFB smears microscopy.

[45] Klarkowski DB, Orozco JD (2010). Microscopy quality control in Médecins Sans Frontières programmes in resource-limited settings. PLoS Med..

[46] Brugha R, Zwi A (1998). Improving the quality of private sector delivery of public health services: challenges and strategies. Health Policy Plan..

[47] Rowe AK, Savigny DD, Lanata CF, Victoria CG (2005). How can we achieve and maintain high-quality performance of health workers in low-resource settings?. Lancet.

